# Bending Behavior of Hybrid Timber–Steel Beams

**DOI:** 10.3390/ma17051164

**Published:** 2024-03-01

**Authors:** Peter Haase, Simon Aurand, Jakob Boretzki, Matthias Albiez, Carmen Sandhaas, Thomas Ummenhofer, Philipp Dietsch

**Affiliations:** 1 Steel and Lightweight Structures, Karlsruhe Institute of Technology, 76131 Karlsruhe, Germany; peter.haase@kit.edu (P.H.); jakob.boretzki@kit.edu (J.B.); thomas.ummenhofer@kit.edu (T.U.); 2 Timber Structures and Building Construction, Karlsruhe Institute of Technology, 76131 Karlsruhe, Germany; simon.aurand@kit.edu (S.A.); carmen.sandhaas@kit.edu (C.S.); dietsch@kit.edu (P.D.)

**Keywords:** timber, steel, hybrid, adhesive bonding, four-point bending, analytical calculations

## Abstract

Driven by climate change and the need for a more sustainable construction sector, policy is increasingly demanding and promoting timber hybrid construction methods. In the German state of Baden-Württemberg, every new public building has to be of timber or timber hybrid construction (Holzbauoffensive BW). The objective of multi-story buildings with large floor spans can only be achieved in a resource-efficient way by hybrid constructions combining timber and steel components. A research project recently completed at the Karlsruhe Institute of Technology was aimed at the development and systematic investigation of hybrid bending beams in which an advantageous combination of the materials steel and timber is used. For this purpose, steel profiles are integrated into timber cross-sections in a shear-resistant manner by adhesive bonding. As part of the experimental, numerical and analytical investigations, different cross-sections of steel and timber, as well as different construction materials, were considered (GL24h, LVL48p, LVL80p, S355 and S420). The results of large-scale four-point bending tests illustrate the potential of this new hybrid construction method. Depending on the geometry and material combinations tested, the bending stiffness could be increased by up to 250%, and the load-carrying capacity by up to 120%, compared to a glulam beam with identical dimensions.

## 1. Introduction

### 1.1. Motivation and Scope of Paper

**Figure 1:**
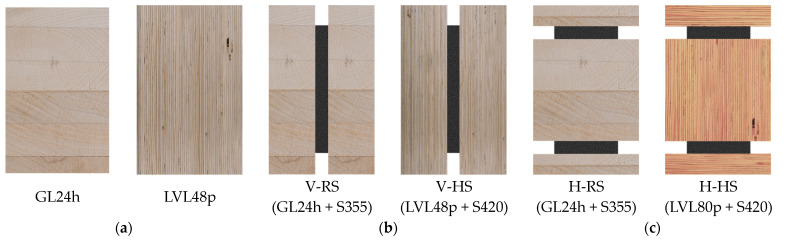
Investigated cross-sections. (**a**) Reference specimens. (**b**) Vertical geometry (V). (**c**) Horizontal geometry (H). (RS = regular strength and HS = higher strength).

Timber structures are becoming increasingly important as an alternative to conventional CO_2_-intensive construction methods. Especially in the area of building classes 4 and 5 (height > 13 m), timber construction projects are increasingly being carried out. Currently the tallest timber construction is the Mjøstårnet high-rise, with a height of 84.5 m [1]. Lighthouse projects such as the Mjøstårnet show that timber structures can also be used at this building scale, but enormous column, beam and bracing cross-sections must be used. The largest column cross-section is 1485 × 625 mm^2^, used for the corner columns of the building. Floors 12 to 18 have concrete floors which are supported by beams with a cross-section up to 625 × 720 mm^2^, and the cross-sectional dimensions of the bracings are up to 625 × 990 mm^2^. In addition to the large cross-sections, the connections are very complex, with up to four parallel slotted-in steel plates and 45 dowels each [1]. The dimensions of the cross-sections illustrate that pure timber constructions also have technical, economic and environmental limitations. A combination of steel components embedded in the timber cross-sections can lead to a considerable reduction of the required cross-sectional dimensions. In a recently finished research project at the Karlsruhe Institute of Technology (KIT), hybrid beams were developed, systematically investigated and optimized towards bending capacity. In Figure 1, all experimentally investigated cross-sections are illustrated. In this work, test specimens with an adhesive bond between the materials are examined. Previous investigations on small-scale hybrid specimens subjected to tension have shown that an adhesive bond between wood and steel achieves a full bond, i.e., the adhesive layer is not decisive for the load-carrying capacity [2].

The scope of this paper is to investigate the influence of different material and geometry combinations on the bending stiffness and the load-carrying capacity. Therefore, two different geometries and three material combinations were experimentally tested. Under the assumption of a full bond, analytical calculations were performed to determine the load-carrying behavior and load-carrying capacity of the test specimens. The results of the analytical calculations were compared with the test results for validation.

### 1.2. State of the Art of Hybrid Timber Construction

Engineered wood products (EWPs) such as glulam and laminated veneer lumber are state-of-the-art in timber construction. Large-scale projects such as Mjøstårnet cannot be built without the use of such products. Additionally, to transfer the high loads, hybrid timber-to-steel connections with slotted-in steel plates had to be used [1]. Numerous research projects investigated timber–timber hybrid cross-sections, for example, hardwood in the outer laminations and softwood in the core of the cross-section [3,4]. Timber–concrete composite components represent another form of hybridization. This technology can be used for refitting old buildings to increase the load-carrying capacity of floor systems. It is also used in new buildings to meet sound insulation requirements [5,6,7].

Timber–steel hybrid construction methods have been part of numerous international research projects for many years. The first articles on this topic were published as early as 1984 [8]. Since then, however, hybrid systems have hardly been able to become established on the market. Riola Parada [9] provides an overview of research work in the field of timber–steel hybrid construction methods for beams. More recent work also focuses on hybrid columns under static and dynamic load [10,11,12,13]. In Sydney, 48 mid-scale (1.2 m) and 22 large-scale (2.0 m) specimens were tested with different material and geometry combinations. The load-carrying capacity was increased up to 107% [14,15].

Long-term tests on hybrid bending beams have shown that the bending stiffness and load-carrying capacity could be significantly increased (3–4 times) compared to pure timber beams. In addition, the long-term tests have shown that the creep behavior could be significantly reduced (approx. 65% less creep after 3 months) in comparison to pure timber beams [16]. The applicability of the elastic theory of layered beams was investigated for timber–steel hybrid beams in which a cross-laminated timber (CLT) was placed on a steel beam. It could be shown that the analytical calculation provides accurate results [17]. Numerical and analytical analyses of steel–timber composite beams with steel girders and LVL timber elements have been performed. Composite beams can be designed analytically assuming a stiff connection method. This shows the importance of the joining technology used [18]. Adhesive bonding is particularly important in this context. While bonding in timber construction has been state-of-the-art for decades [19], bonding in steel construction has been the subject of intensive research in recent years. Both new types of bonded connections [20,21] for steel structures and hybrid joining methods [22], combining grouts and adhesives [23,24] and bolts and adhesives [25,26] are being comprehensively investigated.

## 2. Materials and Methods

### 2.1. Materials

#### 2.1.1. Timber, Wood Products and Steel

The investigated specimens were produced with glued laminated timber GL24h, laminated softwood veneer lumber (LVL48p) and laminated beech veneer lumber (BauBuche LVL80p). The mechanical properties of bending strength, shear strength and Young’s Modulus were determined in accordance with DIN EN 408 [27] and DIN EN 26891 [28]. The test procedure is described in Section 2.2.3. For the mechanical properties of LVL80p, the authors refer to the LVL leaflet [29]. To convert the characteristic values into mean values, a coefficient of variation of 5% was assumed for LVL80p. All timber material properties are listed in Table 1. Two steel grades according to DIN EN 10025 [30] were used to produce the specimens. The Young’s Modulus was assumed with *E* = 210,000 MPa according to DIN EN 1993-1-1 [31]. Tensile tests according to DIN EN ISO 6892-1 [32] were performed to determine the yield strength of the steel products. One mild steel (regular-strength) with a yield strength of *f*_y,RS_ = 380 ± 15.0 MPa (S355) and one higher-strength steel with *f*_y,HS_ = 449 ± 19.8 MPa (S420) were used. For the vertical geometry (V), one steel profile with a height of *h* = 120 mm and a thickness of *t* = 12 mm was used. In the horizontal geometry (H), two steel profiles with a width of *b* = 60 mm and a thickness of *b* = 12 mm were used.

#### 2.1.2. Adhesives

To bond the materials steel and timber, three different adhesives were used. Two bisphenol-A-based two-component epoxy adhesives (subsequently referred to as EP1 and EP2) and one polyurethane (PUR) system were compared. For the material values of EP1, the authors refer to literature [33]. Tensile tests according to DIN EN ISO 527-2 [34] were performed to define the material properties of the EP2 and PUR adhesives. The mechanical properties are shown in Table 1.

### 2.2. Methods

#### 2.2.1. Specimen Geometry and Test Program

**Figure 2:**
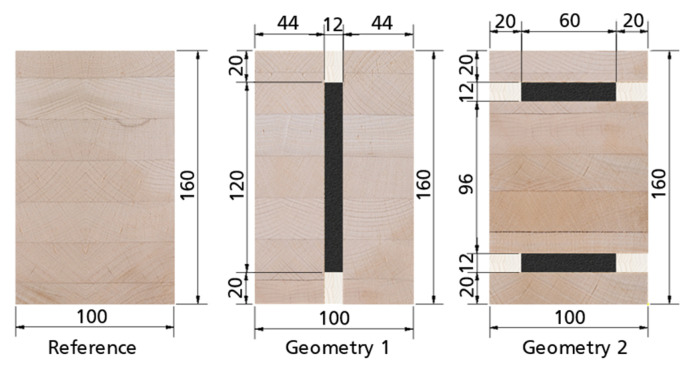
Specimen geometry regular strength (RS) [mm].

The investigated specimens are shown in Figure 2, whereby reference beams of GL24h and LVL48p were tested. Geometry 1 was tested with mild steel + GL24h (regular strength (RS)) and higher-strength steel + LVL48p (higher-strength (HS)). For geometry 2, the HS combination LVL80p was used. The cross-sections in Figure 1 show gaps between the outer timber components. In a later application of the hybrid beams, these gaps are filled with infill timber to ensure fire protection. For the experimental tests, this was not done for manufacturing reasons.

Table 2 shows an overview of the test campaign with hybrid specimens. The steel specimen was either blasted with corundum or galvanized. To identify the specimens, a code is introduced to uniquely describe each specimen. The first letter denotes the orientation of the steel sheet (V–vertical and H–horizontal). The next two letters denote the material combination (RS–regular strength and HS–higher strength). The fourth letter denotes the steel surface (G–galvanized and B–blasted). The last three letters denote the adhesive (EP1, EP2 and PUR) and are followed by a sequential number of the specimen in this series.

#### 2.2.2. Manufacturing of the Specimens

**Figure 3:**
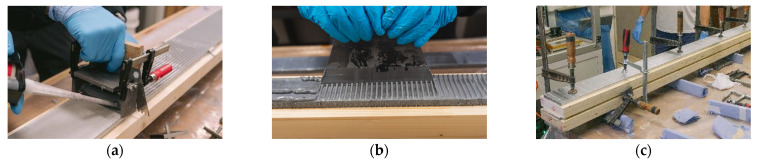
Manufacture of the specimens. (**a**) Distribution of adhesive parallel. (**b**) Distribution of adhesive perpendicular. (**c**) Clamped specimen while curing.

In total, 22 specimens bonded with three different adhesive systems were manufactured: Eleven specimens with geometry 1 and eleven specimens with geometry 2. As shown in a corresponding publication, the surface of the joining parts must be prepared [2]. Therefore, the blasted (SA 2 ½ DIN EN ISO 8501-1 [35]) and the galvanized steel surface were degreased with methyl ethyl ketone (MEK). Additionally, for the PUR adhesive, a primer was required to prepare the steel surface. According to DIN EN 14080 [36], the timber surface was planed within 24 h before manufacture. The adhesive was applied with a static mixer and distributed with a notched spatula with 4 mm notches (see Figure 3a). While curing, the specimens were clamped together with screw clamps. To distribute the force of the screw clamps, an additional steel profile was placed on top and below the specimen (see Figure 3c). After curing, those profiles were removed. Since the open time of the used adhesives is limited, the manufacture time was recorded for all specimens.

**Figure 4:**
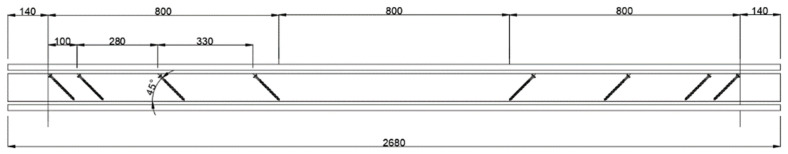
Inclined screws as shear reinforcement [mm].

For the first specimens with EP1 (geometry 1), the adhesive was distributed perpendicularly to the length of the beam (see Figure 3b). This resulted in a manufacture time of 30–35 min, which is the limit for this adhesive system. The distribution parallel to the length of the beam speeds up the manufacture, so that the other specimens could be manufactured within 10–15 min. For the test specimens of geometry 2 with the RS materials, two rows of inclined fully threaded screws (*d* = 8 mm and *l* = 200 mm) were inserted into the middle timber element as shear reinforcement (see Figure 4) [37]. The screws were inserted before the timber element was planed and adhesively bonded. As explained in Section 2.2.3, the load was applied directly to the steel profile. For geometry 2, holes were drilled into the outer timber lamellas before manufacturing. During manufacturing, some adhesive residue and wood fibers remained in the holes. The support of the beam is managed via four steel stamps which are in direct contact with the lower steel plate.

#### 2.2.3. Experimental Testing

**Figure 5:**
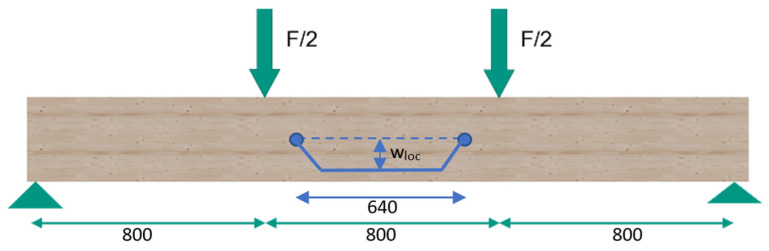
Four-point bending test [mm].

This publication investigates the bending behavior of hybrid timber–steel beams. Therefore, four-point bending tests according to DIN EN 408 were performed with hybrid beams with two different geometry and material combinations, as well as with reference timber beams. Figure 5 shows the test setup. All specimens had a height of 160 mm, as shown in Figure 2. In order to prevent local compression failure perpendicular to the grain of the timber, the load was induced directly into the steel profiles via steel load inlets. All specimens were tested with a span of *l* = 2.4 m.

According to DIN EN 408, the global and local deflections of the beams were measured on both sides of the beam at mid-height. Inductive displacement transducers with a measuring length of 100 mm were used for the global deflection and with a measuring length of 20 mm for the local deflection. The measured values from the front and rear sides were averaged for the evaluation. DIN EN 408 describes the evaluation of the modulus of elasticity (MOE) via the local deflection (w_loc_) by applying the second moment of inertia [27]. This local displacement measurement provides a much more precise picture, as there are no distorting influences from load introduction or the support situation. Since this paper focuses on hybrid bending beams, the bending stiffness (*EI*) is decisive for the comparison. Accordingly, this value was evaluated for every specimen in the range of 10–40% of the ultimate load. Additionally, the steel stresses were recorded with strain gauges. For geometry 1, one strain gauge was applied to each edge fiber (EF) of the steel. In geometry 2, the outer timber was glued to the edge fiber of the steel (see Figure 2), so that the strain gauges were arranged laterally in the center of the steel sheets. All specimens were tested at a speed of 5 mm/min. In contrast to DIN EN 408, the deformation was increased until 60% of the expected load-carrying capacity was reached. Afterwards, the deformation was reduced until the load reached 5 kN in order to remove the local displacement transducers. Afterwards, the deformation was increased constantly until failure (hysteresis loop).

#### 2.2.4. Analytical Calculations

As shown in a corresponding publication, full bonding between timber and steel can be assumed in order to analytically determine the load-carrying capacity [2]. This section describes the evaluations of the hybrid geometries. The ratio of the moduli of elasticity (MOE) (see Section 2.1.1) was used to calculate referred cross-sectional values for both the steel (subsequently indexed *S*) and the timber (index *T*). The second moment of inertia $I_{y,ref,T}$ (with regard to the timber material) and the bending stiffness of the composite section $\bar{EI}$ are calculated with Equations (1) and (2). The second moment of inertia and the bending stiffness of the cross-sections are shown in Table 3.

(1) $$I_{y,ref,T}=\sum I_{y,T,i}+\frac{E_{S}}{E_{T}}\cdot \sum I_{y,S,i}$$

(2) $$\bar{EI}=E_{T}\cdot I_{y,ref,T}=E_{S}\cdot I_{y,ref,S}$$

**Figure 6:**
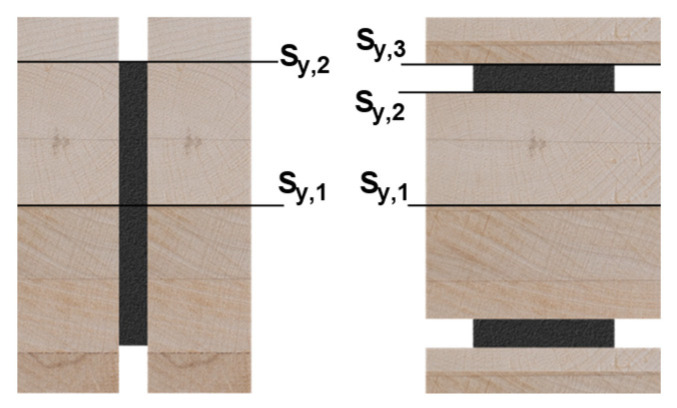
First moment of inertia.

The shear stresses are calculated with the first moment of inertia. As the width of the cross-section is not constant, various points are relevant for the evaluation of the shear stress. Two points (geometry 1) and three points (geometry 2) of the cross section must be evaluated for every cross section (see Figure 6). The calculation of the first moment of inertia is equivalent to the calculation of the second moment of inertia with the corresponding ratio of the MOEs. Since shear failure in the timber becomes decisive, the first moment of inertia is only evaluated with regard to the timber material; see Equation (3). The results are shown in Table 4.

(3) $$S_{y,ref,T}=\sum S_{y,T,i}+\frac{E_{S}}{E_{T}}\cdot \sum S_{y,S,i}$$

With those cross-sectional values, the expected load-carrying capacities for the four-point bending test were calculated. For *S_y_*_,*ref*,1,*T*_ of geometry 1, the width *b* of the steel has to be transferred to an equivalent width of timber by multiplying with the ratio of MOE; see Equation (4).

(4) $$\tau_{T}=\frac{V\cdot S_{y}}{I_{y}\cdot \left(b_{T}+\frac{E_{S}}{E_{T}}\cdot b_{S}\right)}$$

*S_y_*_,*ref*,2,*T*_ and *S_y_*_,*ref*,3,*T*_ were calculated with the width of the steel element. Table 5 in Section 2.2.4 shows the expected load-carrying capacities, with the decisive value marked with (*). Following are the equations to calculate the respective load-carrying capacities.

(5) $$F_{exp,T,m,y}=\frac{6\cdot f_{m,mean}\cdot I_{y,T}\;}{l\cdot \;z_{T}}$$

(6) $$F_{exp,S,y}=\frac{6\cdot f_{y}\cdot I_{y,S}\;}{l\cdot \;z_{S}}$$

(7) $$F_{exp,T,v}=\frac{2\cdot \;f_{v,mean}\cdot I_{y,T}\cdot b}{S_{y}}$$

where

$z_{T}$: Vertical distance to the timber edge fiber

$z_{S}$: Vertical distance to the steel edge fiber

**Table 5:** Expected load-carrying capacity under four-point bending.

	Material	*F_exp_*_,*T*,*m*,*y*_ [kN]	*F_exp_*_,*S*,*y*_ [kN]	*F_exp_*_,*T*,*v*_ [kN]
Geometry 1 (vertical steel plate)	GL24h + S355	68.7 *	52.4	278.9
	LVL48p + S420	95.0 *	66.1	316.1
Geometry 2 (horizontal steel plates)	GL24h + S355	116.8	89.3	71.0 *
	LVL80p + S420	199.0	120.5 *	133.0

* Decisive load-carrying capacity.

## 3. Results

### 3.1. Reference Specimens

**Figure 7:**
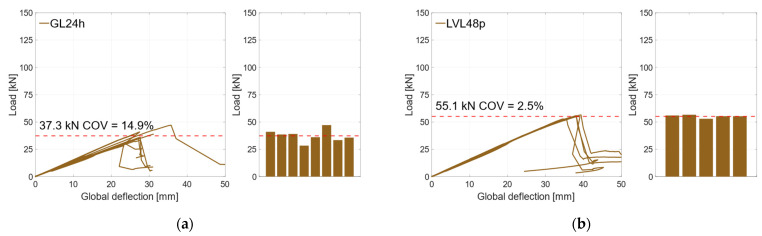
Global behavior. (**a**) Reference specimens RS. (**b**) Reference specimens HS.

To determine the material properties of the used timber materials GL24h (RS) and LVL48p (HS), reference specimens were tested, with *n* = 8 (GL24h) and *n* = 5 (LVL48p). In Figure 7a, the global behavior of the RS-reference specimens is illustrated. As the deflection increased, the load increased linearly until the specimens failed brittle at a mean load-carrying capacity of *F*_max_ = 37.3 kN (*COV* = 14.9%), which corresponds to a bending strength of *f*_m,0,flat,m_ = 35.0 ± 5.2 MPa. It is to be expected that the coefficient of variation decreases with a larger sample size. The bar chart on the right of Figure 7a highlights the differences between the maximum loads of the specimens. The stiffness *EI* and the MOEs were evaluated according to DIN EN 408, with the local deformation between 10% and 40% of *F*_max_. The RS specimens reached a stiffness of *EI* = 37.7 × 10^10^ Nmm^2^, which corresponds to a modulus of elasticity of *E* = 11,100 MPa for the given rectangular cross-section. Figure 7b) illustrates the reference specimens with HS materials. Here, all specimens showed a very similar linear behavior until the specimens failed in a brittle manner under bending stress at *F*_max_ = 55.1 kN (*COV* = 2.5%), which corresponds to a bending strength of *f*_m,0,flat,m_ = 51.7 ± 1.3 MPa. The local bending stiffness was evaluated as *EI* = 43.0 × 10^10^ Nmm^2^, which corresponds to a Young’s Modulus of *E* = 12,600 MPa.

### 3.2. Geometry 1 (Vertical Steel Sheet)

#### 3.2.1. Material Combination: Regular Strength (RS)

**Figure 8:**
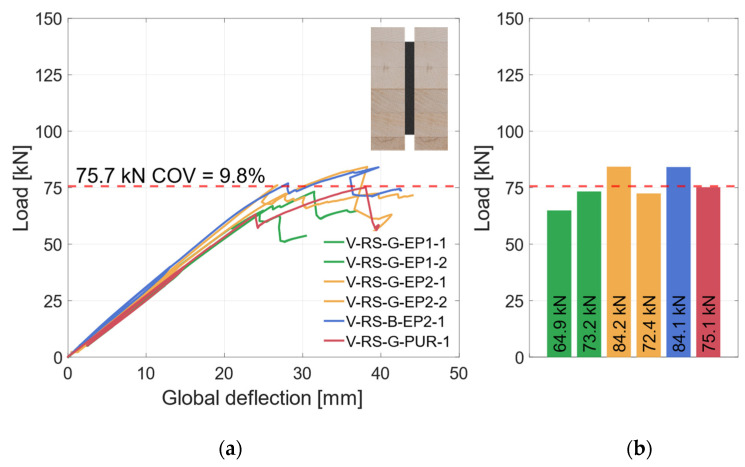
Global behavior—specimens with vertical steel and GL24h + S355 (RS). (**a**) Global load–deflection. (**b**) Load-carrying capacity.

**Figure 9:**
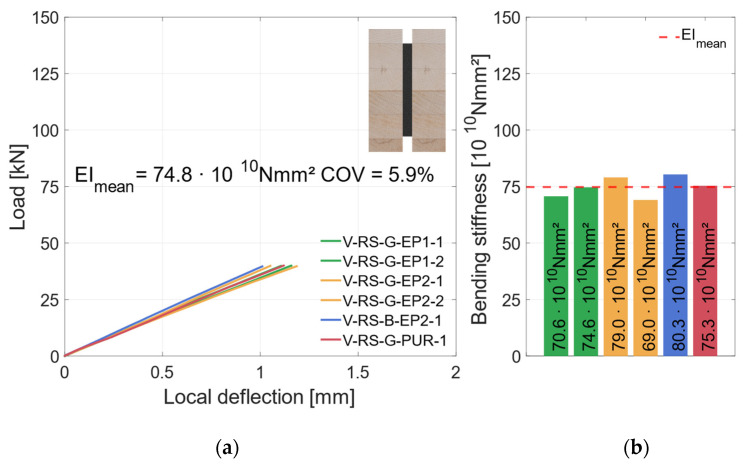
Local behavior—specimens with vertical steel and GL24h + S355. (**a**) Local load–deflection diagram. (**b**) Bending stiffness.

Figure 8a shows the global load–deflection diagram of all specimens with a vertical steel sheet and RS materials. As explained in Section 2.2.3, the local and global deflections were measured on both sides of the specimens. During the first loading up to 45 kN, the global load–deflection behavior is almost linear. The same applies for the local deflection that is plotted in Figure 9a. To improve readability, the diagram in Figure 9a is cut off at 0.6 × *F*_est_, which marks the beginning of the hysteresis loop. Since local effects and shear stress due to shear force influence the global deflection measurement, the bending stiffness is evaluated on the basis of the local deflection measurement; see Figure 9b. As all test specimens exhibit very similar load–deflection behavior, the behavior is explained using an example beam (V-RS-G-EP2-1). The hysteresis loop induces no additional deflection. Upon reloading, the load rises linearly until a first load drop occurs at about 75 kN. At this point, small cracks appear in the timber elements. After this load drop of approx. 5 kN, the load increases again, yet with gradually decreasing stiffness, until a second load drop occurs at the maximum load of 84.2 kN. The load drops by approximately 20 kN. Then the deflection is increased with negligible change in load until the test was stopped.

**Figure 10:**
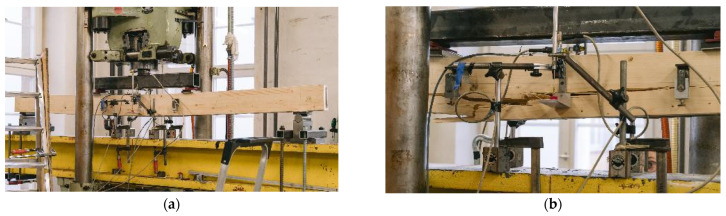
Experimental investigations of specimen V-RS-G-EP2-1. (**a**) In the testing machine during testing. (**b**) After failure.

**Figure 11:**
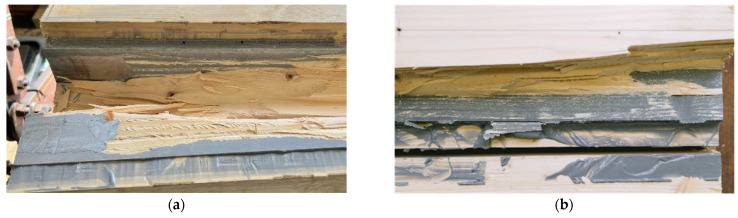
Fracture patterns. (**a**) Partial failure in the timber and in the zinc coating V-RS-G-EP2-2. (**b**) Wood fibers on the adhesive V-RS-B-EP2-1.

Figure 10a shows a specimen in the testing machine during the test, Figure 10b displays the specimen after its second load drop. The cracks in the timber element indicate that the specimen fails due to tensile bending. As the number of test specimens in each series is small, no statistical evaluation regarding the influence of the adhesive or the steel surface is made. The graph in Figure 8a shows that all specimens exhibited very similar behavior prior to the first load drop. The initial load drop was due to tensile bending failure in the timber element. Afterwards, for some of the specimens with a galvanized steel surface, a partial delamination in the zinc layer (Figure 11a) occurs. Figure 11b shows the fracture pattern of a specimen with a blasted steel surface. Here the failure is entirely in the timber element, with wood fibers remaining on the adhesive. The differences in behavior after the initial failure are probably caused by the variation in the mechanical properties of the timber. As all test specimens exhibit very similar behavior; they are grouped as “vertical—regular strength” (V-RS) in the following with *EI* = 74.8 × 10^10^ Nmm^2^ (*COV* = 5.9%), shown as red dashed line in Figure 9b. The mean load-carrying capacity of this group is *F*_max_ = 75.7 kN (*COV* = 9.8%), as shown in Figure 8a as a red dashed line. As explained in Section 2.2.2, two different methods of adhesive application were investigated. The adhesive was applied perpendicularly to test specimen V-NF-G-EP2-1. For the other test specimens, the adhesive was applied longitudinally. The results show that the direction of application did not significantly affect the load-carrying capacity and stiffness of the test specimens. However, when the test specimens were opened, the ridges of the adhesive application (see Figure 3b) were partially visible. This indicates that the adhesive had already started to cure during the application time of 30–35 min.

**Figure 12:**
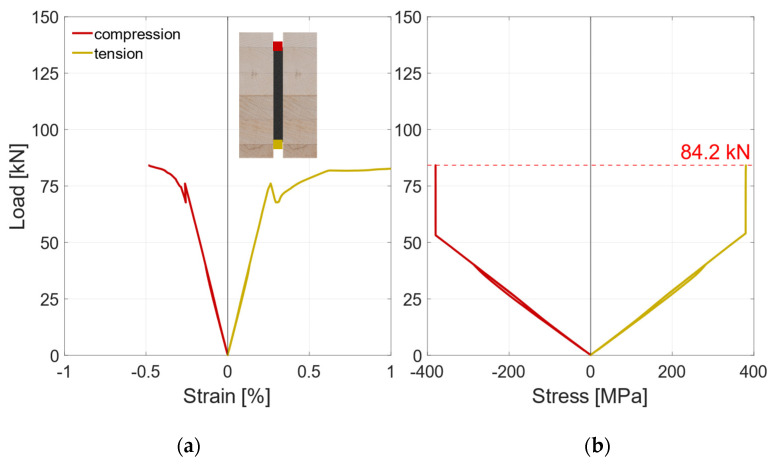
V-RS-G-EP2-1 Strain gauges. (**a**) Load–strain diagram. (**b**) Load–stress diagram.

Besides the local and the global deflection, the steel stresses were measured with two strain gauges. The strain gauges are marked red and yellow in the pictogram in Figure 12a, which shows the results of the strain gauges of an exemplary test specimen. The strain rises linearly up to the first load drop at approx. 75 kN. After this, a load transfer to the steel profile can be observed. As the edge fibers of the steel are already in the plastic range, the strains increase substantially under a constant load until the second load drop occurs. This indicates full plastic utilization of the steel profile. In Figure 12b, the load–stress diagram is illustrated. The stress is calculated assuming a linear elastic ideal plastic behavior. It indicates that at loads above 50 kN, the maximum elastic strain determined in tensile tests (see Section 2.1.1) is exceeded at the upper and lower edge of the steel profile. Upon reaching the maximum load, approximately 40% of the steel’s cross-section is in the plastic range.

#### 3.2.2. Material Combination: Higher Strength (HS)

**Figure 13:**
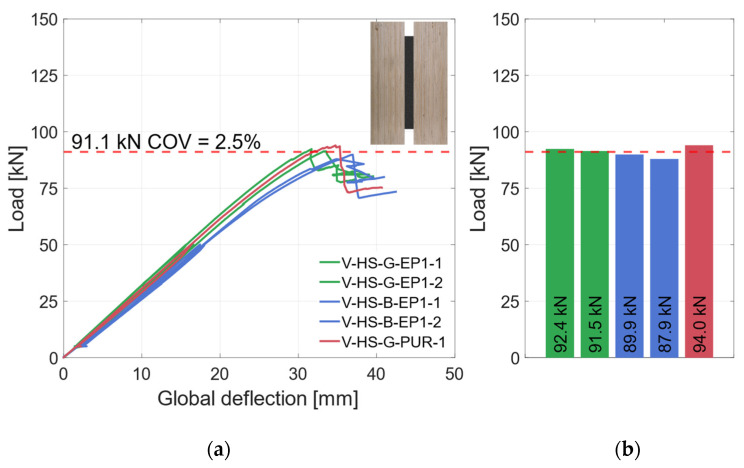
Global behavior—specimens with vertical steel and LVL48p + S420. (**a**) Global load–deflection. (**b**) Load-carrying capacity of the HS specimens.

**Figure 14:**
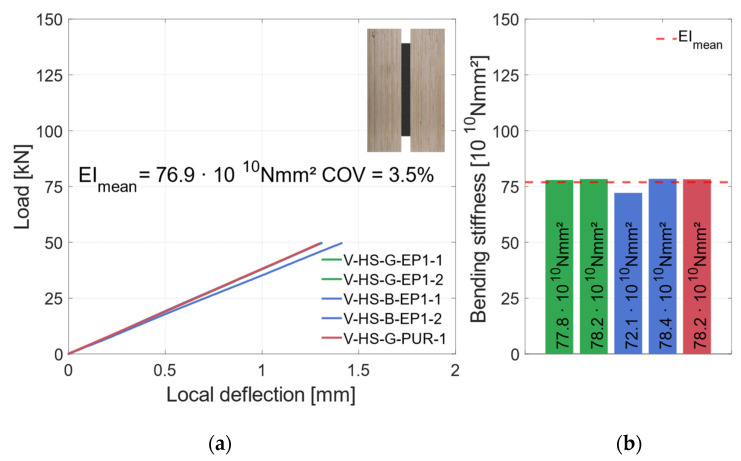
Local behavior—specimens with vertical steel and LVL48p + S420. (**a**) Local load–deflection diagram. (**b**) Bending stiffness.

The global and local load–deflection behavior of all tested specimens with vertical steel plates and the materials LVL48p and S420 are shown in Figure 13a and Figure 14a. The global behavior in Figure 13a demonstrates very similar behavior of the specimens with higher-strength materials. There is no significant difference between the blasted and galvanized surface preparations. With increasing deflections, the load rises linearly until the specimens fail due to tensile bending in the timber element. The ultimate load of the specimens in this test series is shown in Figure 13b. Averaged, the specimens reached *F*_max_ = 91.1 kN (*COV* = 2.5%), as shown as a red dashed line in Figure 13a,b.

The small coefficient of variation confirms the homogenization effect of the LVL in combination with the steel. After a small load drop, the load rises again, but accompanied by large deflection, and thus never reaches the first maximum again. The fracture patterns of the different specimens show a similar failure as well. The initial failure of all specimens was tensile bending in the timber element. The results of the strain gauges showed that the higher-strength steel could also be partially plastically utilized. The local behavior (see Figure 14a) is also very similar for all specimens (*COV* = 3.5%). It should be mentioned that specimen V-HS-B-EP1-1 was not as stiff as the other specimens in this category (see Figure 14b). This is probably due to the variation of the mechanical properties of the timber. Nevertheless, the local and global behaviors of all specimens in this category are very similar. In the following all specimens will be summarized in one group “vertical—higher strength” (V-HS).

### 3.3. Geometry 2 (Horizontal)

#### 3.3.1. Material Combination: Regular Strength (RS)

**Figure 15:**
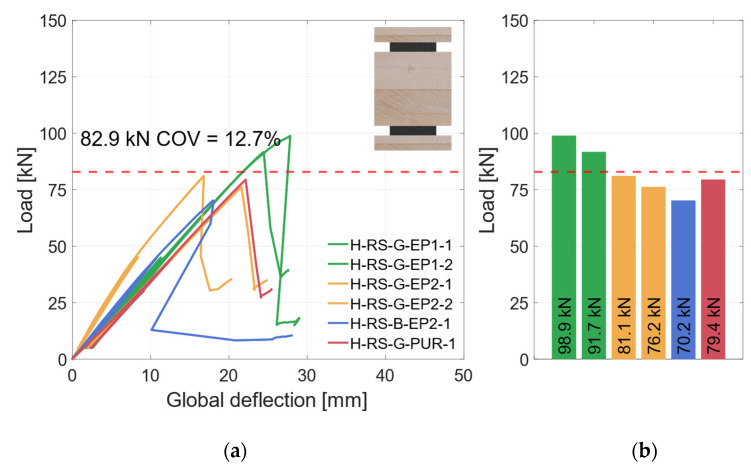
Global behavior—specimens with horizontal steel and GL24h + S355. (**a**) Global load–deflection diagram. (**b**) Load-carrying capacity of the RS specimens.

In Figure 15a, the global deflection behavior of the specimens with geometry 2 are shown. With increasing deflection, the load rises linearly up to 50 kN without an influence of the hysteresis loop. Upon reloading, the load then increases linearly again, but with a lower stiffness until the specimens fail brittle at *F*_max_. The failure is accompanied by a decisive load drop. Afterwards, the deflections were increased further. The load rises again but with a noticeably lower stiffness until the test was stopped.

As the deformations in the area of the support are included in the global deflection, a quantitative evaluation of the global deflection is inexpedient. All specimens show a linear increase of the load until they fail brittle with a distinct load drop. The load–deflection behavior in Figure 15a differs due to the specific load introduction to the specimen as detailed in Section 2.2.2. Since adhesive and timber fiber residues in the load introduction holes could not be completely eliminated, a minor influence on the global bending stiffness can be observed.

**Figure 16:**
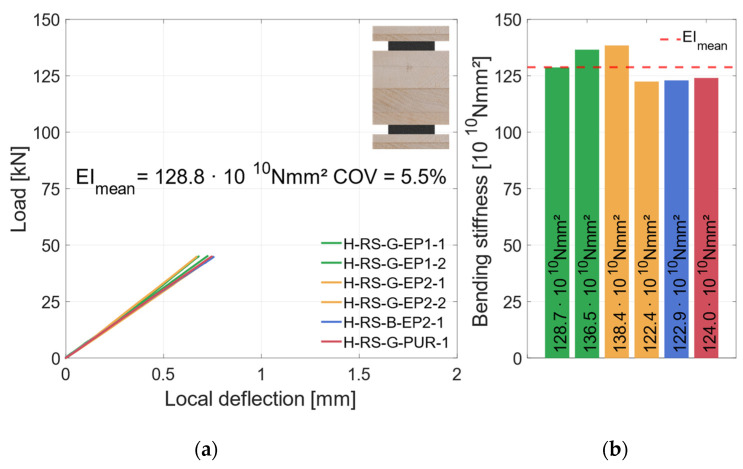
Local behavior—specimens with horizontal steel and GL24h + S355. (**a**) Local load–deflection diagram. (**b**) Bending stiffness.

In Figure 16a, the local behavior is shown. Here, the effects of the support conditions are not included. This shows that the stiffnesses of all specimens were similar. All specimens exhibit a linear load–deflection behavior up to 50 kN, when the local inductive displacement transducers were removed. The bending stiffness values of the specimens in this category vary between 122 × 10^10^ Nmm^2^ and 138 × 10^10^ Nmm^2^, as shown in Figure 16b. The failure mode of all specimens is shear failure in the timber element. The greater differences in load-carrying capacity are due to this failure mode. The measured coefficient of variation of *COV* = 12% is within the expected range of *COV* ≤ 15% of the shear strength [38]. As the number of specimens for each adhesive is low, no statistical evaluation regarding the influence of the adhesive or the steel surface is made. In order to compare specimen groups, all specimens in this category are grouped as “horizontal—regular-strength” (H-RS) subsequently.

**Figure 17:**
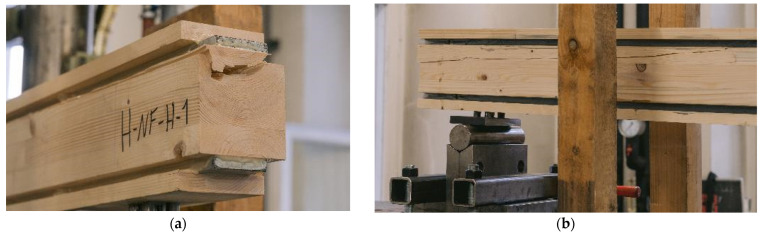
Experimental investigations. (**a**) H-RS-G-PUR-1 shear failure. (**b**) H-RS-G-EP2-2 after failure.

**Figure 18:**
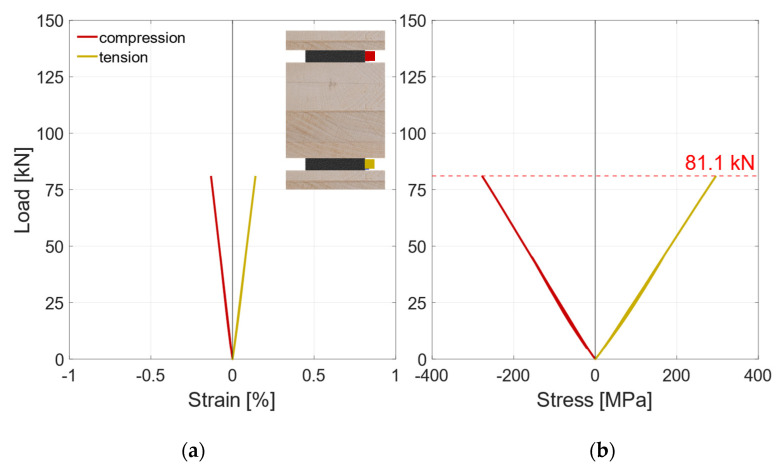
H-RS-G-EP1-2 Strain gauges. (**a**) Load–strain diagram (**b**) Load–stress diagram.

In Figure 17a, the end of the specimen H-RS-G-PUR-1 is shown. The failure pattern shows that the adhesive layer does not become decisive and the middle timber fails in shear. Figure 17b shows another adhesive but a similar failure. The failure starts at the end of the beam and the crack moves along the length of the beam closer to the steel profile. The recorded strain gauge data are shown in Figure 18a for an exemplary test specimen. The strain gauges used had a width of 2 mm and were attached at half the height of the steel. The strain rises linearly until the specimen fails at a strain of approx. 0.14%. In Figure 18b, the stress is evaluated. A strain of 0.14% corresponds approximately to a stress of 295 MPa. As the measured strain is not the strain in the edge fiber of the steel, the maximum strain has to be calculated. According to the Bernoulli hypothesis of the evenness of cross-sections, the strain and the stress in the edge fiber of the steel and the timber can be calculated. This results in a strain of 0.16%, which equals 327 MPa in the edge fiber of the steel. This indicates that the yield strength of the steel has not been reached in the edge fibers.

#### 3.3.2. Material Combination: Higher Strength (HS)

**Figure 19:**
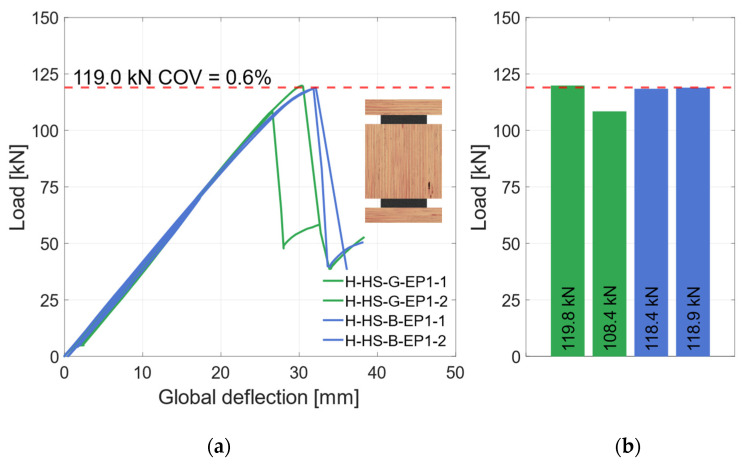
Global behavior—specimens with horizontal steel and LVL80p + S420. (**a**) Global load–deflection. (**b**) load-carrying capacity of the HS specimens.

**Figure 20:**
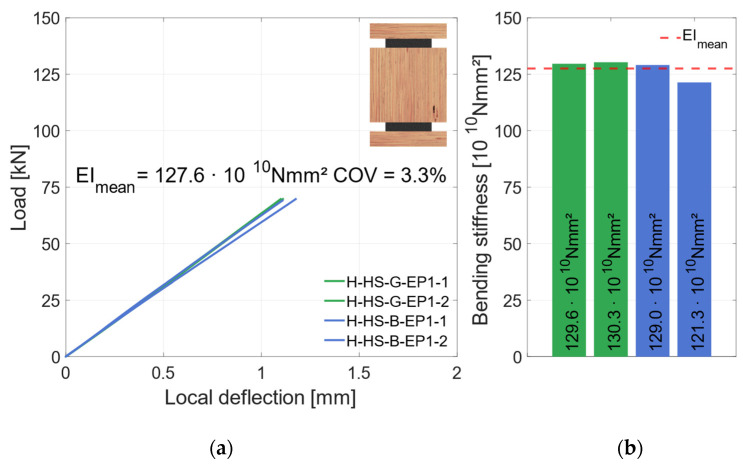
Local behavior—specimens with horizontal steel and LVL80p + S420. (**a**) Local load–deflection diagram. (**b**) Bending stiffness.

Four specimens with EP1, two with a galvanized and two with a blasted surface, were produced and tested. Figure 19 shows the global behavior of those specimens. As shown, three of the four specimens had a very similar behavior. With increasing deflection, the load rises linearly until the specimens fail brittle with a significant load drop. The fracture patterns of those specimens showed a shear failure in the middle timber element. Specimen H-HS-G-EP1-2 had a different failure mode. Here, an adhesion failure between the top steel and the middle timber element occurred in the zinc layer. That can be traced back to local bond weaknesses of the zinc coating and the steel surface. No failure in the adhesive layer or in the adhesive bond between adhesive and zinc layer was observed. Therefore, this specimen was not considered for the mean load-carrying capacity. As shown in Figure 20, the local behavior was not influenced by the different failure mode. Here, all four specimens show a very similar behavior. Hence, all specimens are considered regarding their bending stiffness. The specimens are subsequently grouped as “horizontal—higher strength” (H-HS).

**Figure 21:**
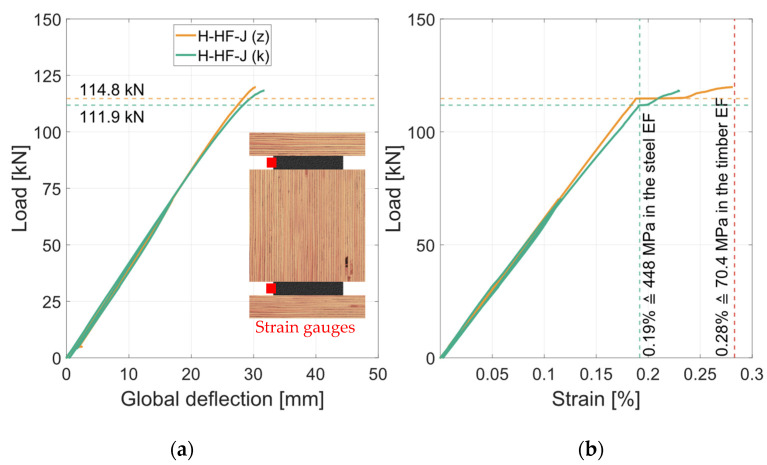
Global load–deflection—steel strain. (**a**) Load–strain diagram. (**b**) Load–stress diagram.

**Figure 22:**
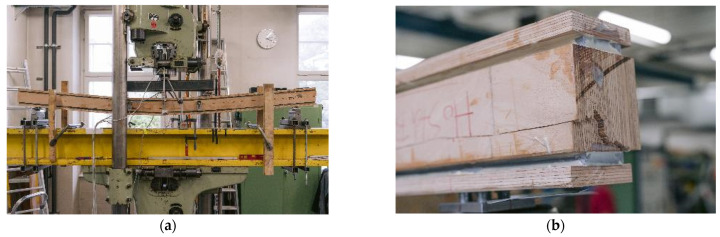
Experimental investigations of specimen H-HS-B-EP1-1. (**a**) In the testing machine after failure. (**b**) Shear failure at the end of the beam.

In contrast to the geometry 2 specimens with RS materials, the steel used for the HS specimens reached its yield stress. Figure 21a shows the global load–deflection diagram and Figure 21b shows the load strain diagram of the lower strain gauge (in the tension zone). Shortly before the failure of the cross-sections, the strains at constant load increase sharply, which indicates a fully plastic utilization of the steel profiles. This indicates that the yield strength of this steel section was slightly lower than 449 MPa, but within the standard deviation (see Section 2.1.1). Reaching this point, the stiffness of the global deflection decreases slightly and the specimens fail brittle in the inner timber element due to shear failure. As LVL80p with a bending strength of *f*_m,y,k_ = 80 MPa [21] was used, and the maximum tension stress in the edge fiber is calculated to 70.4 MPa, no failure occurred in the outer timber lamella. Figure 22a shows this failure mode with a still functioning outer lamella with very large deformations. The shear failure of this specimen is illustrated in Figure 22b.

Due to the test specimen size and the number of investigated parameters, the sample quantity in each test series was small (2–3 per series). Therefore, no statistical evaluation was conducted to analyze the significance of the investigated parameters.

## 4. Discussion

This section highlights and discusses the differences between the individual specimen groups (V-RS, V-HS, H-RS and H-HS). In the first step, the different material combinations for geometry 1 and geometry 2 are compared. The geometry combinations are then compared for the RS and the HS materials combined with the reference specimens. In the last step, the experimental results are compared to the analytical investigations.

### 4.1. Influence of the Material Combinations

#### 4.1.1. Vertical Geometry

**Figure 23:**
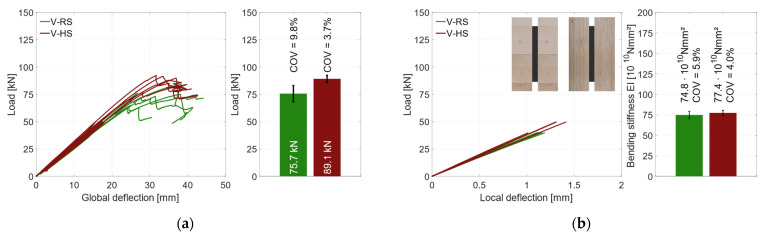
Vertical regular strength (V-RS)—higher strength (V-HS). (**a**) Global behavior *F*_max_ [kN]. (**b**) Local behavior *EI* [Nmm^2^].

To compare the material combinations, Figure 23a shows the global behavior and the mean load-carrying capacity. All specimens with regular-strength (RS) materials are illustrated in green, and the higher-strength (HS) materials are illustrated in red. Regardless of the material used, all specimens had a very similar global behavior up to about 50 kN and a global deformation of 20 mm. Afterwards the specimens with HS materials were slightly stiffer. As explained in Section 3.1, the RS specimens showed a small load drop before *F*_max_ was reached. The RS specimens reached a mean load-carrying capacity of *F*_max_ = 75.7 kN, with a coefficient of variation of *COV* = 9.8%. With HS materials, there was no load drop before *F*_max_ was reached. The failure is ductile without a distinct load drop. A mean load-carrying capacity of 89.1 kN (*COV* = 3.7%) was measured. The higher-strength materials were able to increase the load-carrying capacity by 18%. In addition, the coefficient of variation for the HS materials is substantially lower than for the RS materials, which is assumed to be due to the lower scatter of the mechanical properties of the LVL. The local behavior is shown in Figure 23a. There was no significant difference between the bending stiffness values (only 3.4%) since the coefficients of variation were 5.9% (RS) and 4.0% (HS). This is due to the small stiffness difference between GL24h and LVL48p. For all specimens, the yield strength of the steel profiles was reached, and they showed a ductile behavior after the failure, without a distinct loss of load-carrying capacity. Even with large deformations after the initial failure, no stability failure of the steel could be determined, and the residual load-carrying capacity was maintained.

#### 4.1.2. Horizontal Geometry

**Figure 24:**
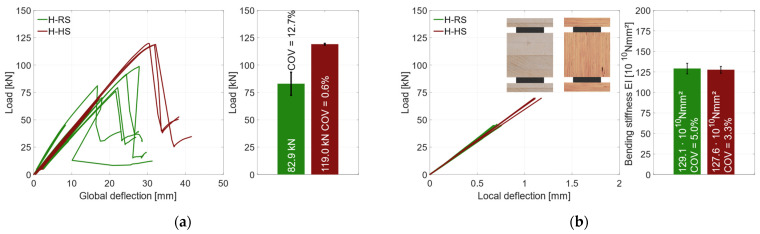
Vertical regular strength—higher strength. (**a**) Global behavior *F*_max_ [kN]. (**b**) Local behavior *EI* [Nmm^2^].

The influence of the used materials on the load-carrying capacity is substantial for geometry 2. Figure 24a shows the global behavior of those specimens, where the failure and post-failure behavior can be analyzed. All specimens, regardless of the material used, fail brittle with a decisive load drop. The specimens with RS materials failed due to shear failure of the middle timber element. With the HS materials, the same behavior occurred. However, the plastic load-carrying capacity of the steel profiles was reached in the horizontal test specimens with HS materials, which is assumed to have caused premature shear failure in the middle timber. The HS materials had a 44% higher load-carrying capacity (*F*_max_ = 119 kN) than the RS materials (*F*_max_ = 82.9 kN). In terms of the coefficient of variation, the influence of the higher-strength materials is even clearer with COV = 0.6% (HS) and COV = 13% (RS). This significant difference can be explained by the different failure modes. Since the steel profiles used for the HS test specimens had a very similar yield strength, the fully plastic moment was also achieved with a very similar load. The subsequent load redistribution could not be carried by the middle timber. In addition, the properties of the beech LVL scatter significantly less than those of the softwood glulam. The evaluation of the local bending stiffness did not reveal any significant differences for the two material combinations, as shown in Figure 24b. Since the steel components are located in the outer areas of the overall cross-section, a large part of the bending stiffness results from the Steiner components. As a result, the higher modulus of elasticity of the beech LVL had no significant influence on the overall stiffness of the hybrid cross-section.

### 4.2. Influence of the Geometries

#### 4.2.1. Regular-Strength Materials

**Figure 25:**
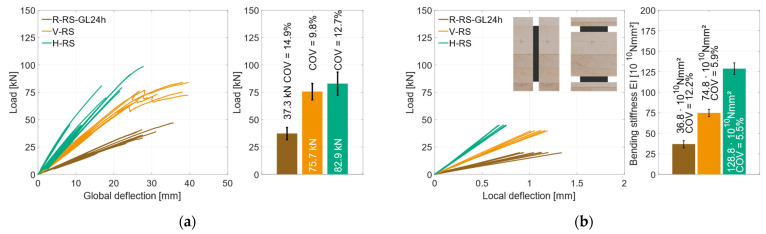
Regular strength reference—vertical—horizontal. (**a**) Global behavior *F*_max_ [kN]. (**b**) Local behavior *EI* [Nmm^2^].

To assess the influence of the different geometry combinations, Figure 25a shows the global behavior of all specimens with RS materials. The vertical specimens reached *F*_max_ = 75.7 kN with *COV* = 9.8%, which is an increase of 100% compared to the reference beams. The horizontal geometry reached *F*_max_ = 82.9 kN with *COV* = 12.7%. In comparison to the reference specimens, this is an increase of 120%. The coefficient of variation is much lower for the specimens of geometry 1 but shows only a minor decrease for geometry 2. Since the specimens with geometry 2 had a different failure mode, the coefficient of variation is not comparable to that of geometry 1 and the reference specimens. The lower COV of the specimens with geometry 1 in comparison to the reference specimens is due to homogenization effect of the steel, allowing the load to be redistributed into the steel in the event of local failure in the timber.

In Figure 25b, the local behavior of those three specimen geometries is illustrated. The bending stiffness of the geometry 1 specimens is 100% higher compared to the reference specimens. This means that a pure timber beam with the same width would have to be 25% higher to achieve the same bending stiffness. With an increase of 250%, the geometry 2 specimens showed an even stiffer behavior compared to the reference specimens. Here, a pure timber beam with the same width would have to be 50% higher. Both geometry combinations result in a decrease of over 50% in terms of stiffness *COV*. This behavior can be attributed to the use of the same amount of steel for both cross-sections, which homogenized the natural scattering of the timber.

#### 4.2.2. Higher-Strength Materials

In the group of higher-strength materials, the comparison is more complex, due to the different timber grades. No reference tests were carried out for beech LVL. Therefore, analytical calculations (noted with A) of an LVL80p beam are used in the following comparison. The minimum coefficient of variation (*COV* = 5%) according to DIN EN 14358 [39] was used to convert the characteristic material values into mean values.

**Figure 26:**
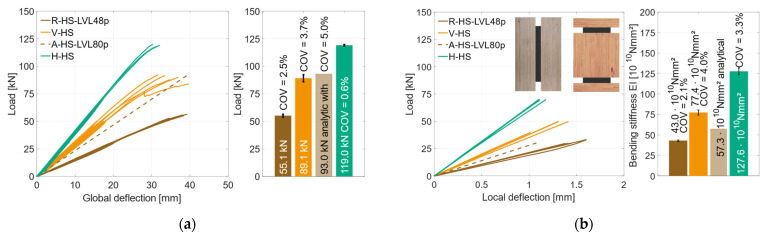
Higher-strength reference—vertical—horizontal (**a**) Global behavior *F*_max_ [kN] (**b**) Local behavior *EI* [Nmm^2^].

Figure 26a shows the global behavior of all specimens with higher-strength materials. The geometry 1 specimens reached *F_max_* = 89.1 kN (*COV* = 3.7%) which is an increase of 60% compared to the reference specimens with LVL48p. The results show that the coefficient of variation could not be increased by the additional steel. Both tested series showed a very small COV of under 5%. Geometry 2 reached *F_max_* = 119 kN (*COV* = 0.6%) which is an increase of 30% compared to the LVL80p calculations. Due to the small number of test specimens, no statistically verifiable statement can be provided about the coefficient of variation of this test series. To assess the bending stiffness, Figure 26b shows the local behavior of all specimens with HS materials. The specimens with geometry 1 reached *EI* = 77.4 × 10^10^ Nmm^2^ (*COV* = 4.0%), which is an increase of 80% compared to the LVL48p specimens. A pure LVL48p beam with the same width has to be 20% higher. Geometry 2 showed an *EI* = 127 × 10^10^ Nmm^2^ (*COV* = 3.6%), which is an increase of 120% compared to the LVL80p specimens. Here, a LVL80p beam with the same width has to be 30% higher. The coefficient of variation of all tested HS series is very small (≤4.0%).

### 4.3. Comparison of Experimental and Analytical Results

The analytical calculations are shown in Section 2.2.4. The results of the reference specimens were used to calculate the expected load-carrying capacities. In Table 5, the results of the analytical calculations are shown. For the shear failure, the governing value is shown. For geometry 1, the point of *S_y_*_,*ref*,1,*T*_, and for geometry 2, the point of *S_y_*_,*ref*,2,*T*_ is critical, independent of the materials used.

Analytically, reaching the yield strength of the steel is governing for all geometry and material combinations, except for geometry 2, with GL24h and S355. According to the Bernoulli hypothesis, the cross-section remains flat even after the yield strength of the steel has been exceeded. Therefore, with partial plastic utilization of the steel, the deflection could be further increased until the flexural strength of the wood is reached. Therefore, *F_exp_*_,*T*,*m*,*y*_ is marked as the critical load-carrying capacity for geometry 1 in Table 5.

**Figure 27:**
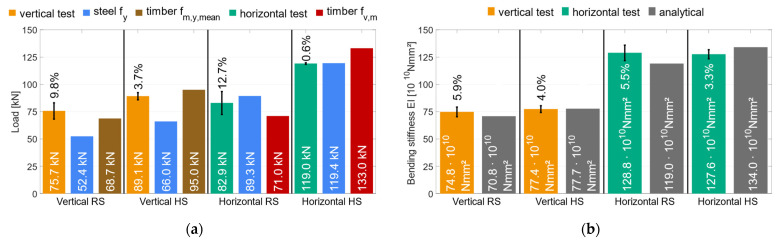
Experimental investigations—analytical calculations. (**a**) Ultimate load *F*_max_ [kN]. (**b**) Bending stiffness *EI* [Nmm^2^].

To compensate for the low shear strength and force tensile bending failure in the experimental tests, inclined screws with an angle of 45° were inserted in the center timber element. Dietsch [37] investigated the influence of inclined screws as shear reinforcement. Accordingly, an increase in shear strength of up to 20% is possible. Due to the inclined screws, the horizontal specimens with RS materials showed an increase in shear strength of 16%. No inclined screws were used with HS materials due to the higher shear strength of LVL80p. In order to compare the analytical calculations with the test results, Figure 27a shows the test results and the decisive analytical load-carrying capacities. The specimens with geometry 1 failed due to tensile bending in the timber elements. The strain gauges showed that the yield strength of the steel was reached before tensile bending occurred, confirming the assumption of plastically utilizing the steel profile. The test specimens were able to exceed the analytical load-carrying capacity (reaching of *f*_m,mean_ in the timber edge fiber) by 13%. According to the analytical calculations, the load of the tests corresponds to a partial plastic utilization of the steel of approx. 35% and a stress in the wood edge fiber of 38.6 MPa. The steel profile homogenizes the inhomogeneous timber and can compensate for local defects by redistributing the load into the steel. Therefore, a local failure in the timber does not lead to a global failure of the hybrid beam. This effect is confirmed by the global load-deformation diagrams (Figure 8). The first load drop before the ultimate load was reached did not lead to a global failure of the specimen. Geometry 1 specimens with HS materials did not reach the bending strength of LVL48p (−6%), but still exceeded the yield strength (+35%). This equals a partially plastic utilization of approx. 40%. Since the LVL is already substantially more homogeneous (*COV* = 2.5% for the LVL48p reference beams), the steel cannot homogenize the cross-section any further, and the first timber failure leads to global failure of the cross-section (see Figure 13).

Figure 27b shows a comparison between experimentally determined (test) bending stiffnesses and analytically calculated bending stiffnesses. The tested and calculated results match well (+5.9% RS and −0.4% HS) for the vertical combination. For the RS materials, the steel is expected to homogenize the timber, which may result in exceeding the analytically calculated load-bearing capacity (+5.9%). The stiffness of HS materials cannot increase (+3.4%) compared to RS materials due to the homogenizing steel. The experimentally determined stiffness exceeds the analytically calculated stiffness (+8.2%) in terms of the horizontal combination, possibly due to manufacturing imperfections and homogenizing effects. The high-strength materials cannot achieve the analytically calculated stiffness (−4.8%). The calculation only used a normative stiffness value for the high-strength timber (BauBuche) due to the absence of reference beams. As with the vertical combination, the use of HS materials cannot further increase the stiffness (−0.9%) of the horizontal combination.

Geometry 2 with RS materials did not reach the yield strength of the steel used. The max steel stress is calculated to 353 MPa in the edge fiber. The shear strength of the timber was exceeded by 14%, which is probably due to the inclined screws in the middle timber element. Beech LVL with a characteristic shear strength of *f_v_*_,*k*_ = 8 MPa was used for the HS test specimens with geometry 2. The characteristic values were converted to mean values with a coefficient of variation of *COV* = 5%. The shear strength could not be reached in the tests (−11%). The yield strength of the HS steel was reached (−0.3%). After the yield strength in the steel was reached, the strains in the steel increased exponentially and the timber is strained accordingly due to the adhesive bond. As Figure 21 shows, the bending tensile strength of the outer timber element is not reached (σ = 70 MPa) and the middle timber element fails in shear.

The previous comparison has shown that the tested beams can be calculated very well using analytical methods. However, this only applies to the analyzed geometry combinations. In an ongoing numerical and experimental investigation, a minimum thickness for the steel profiles must be determined.

## 5. Conclusions

Four-point bending tests on hybrid timber–steel beams were performed to investigate the influence of different geometries and material combinations on the load-carrying behavior. Therefore, reference timber specimens, a vertical combination with one vertical steel profile between two timber elements, and a horizontal combination with two horizontal steel profiles in the outer cross section part were tested. The materials GL24h, LVL48p, LVL80p, S355 and S420 were used to manufacture the specimens. The combination of GL24h and S355 is classified as regular-strength (RS). The higher-strength steel S420 has been combined with LVL48p or LVL80p. These combinations are defined as higher-strength (HS). Subsequently, the main results are summarized; all comparisons are referred to the timber reference specimens with the corresponding timber grade:

- An extensive analytical parameter study showed that the materials timber and steel can be combined beneficially, but that the corresponding material grades must be matched as best as possible in order to utilize the full potential of both materials.
- The vertical combination with regular-strength (RS) materials could increase the bending stiffness by 100%; with higher-strength (HS) materials, the additional steel increased the bending stiffness by 80%.
- All specimens with the vertical geometry combination failed by tensile bending failure in the timber element with a ductile residual behavior. The steel elements could be partially stressed plastically.
- In terms of load-carrying capacity, the specimens with vertical steel and RS materials showed an increase of 100%, and with HS materials, the load-carrying capacity was increased by 60%.
- The horizontal combination with RS materials could increase the bending stiffness by 250%; with HS materials, the additional steel increased the bending stiffness by 120%.
- All specimens with the horizontal combination failed due to shear in the middle timber element. The RS material specimens could not reach the yield strength of the steel. With HS materials, the steel was used fully plastically before shear failure occurred.
- Due to the hybridization, the load-carrying capacity of the horizontal combination was increased by 120% with RS materials and by 30% with HS materials.
- For both geometry combinations, the bending stiffness could not be further increased when using HS materials, compared to using RS materials (see Figure 27b).
- The comparison between the analytical calculations and the experimental investigations confirmed the assumption of a full bond between the materials. Both the bending stiffness and the stress distribution in the cross-section could reliably be calculated analytically.

These results of increased bending stiffness values are particularly relevant, because in conventional timber construction, deflection limits usually dominate the required beam cross-section for bending stress. Due to the significant stiffness increase, timber hybrid constructions open up new areas of application as a resource-efficient alternative to conventional construction methods. Compared to pure steel or timber construction methods, hybridization offers other advantages, such as the applicability and full use of slender steel profiles of cross-section class 4. Since these are elastically bedded by the timber, local stability failure can be prevented before the yield strength is reached in the compressively stressed cross-section parts. There are also significant advantages concerning steel structures in terms of fire resistance, as the timber surrounding the steel section protects it from the effects of fire and rapid temperature rise. In addition, the complex and expensive connection details required to achieve the necessary stiffness compared to conventional timber construction methods can be avoided, as the hybrid construction method allows the use of typical bolted steel connections.

## Figures and Tables

**Table 1:** Material properties (values for EP1 from [33].)

	Material	E-Modulus [MPa]	Bending Strength *f*_m,0,flat,m_ [MPa]	Shear Strength *f*_v,m_ [MPa]	Density *ρ* [kg/m³]	Moisture Content *u* [%]	Tensile Strength *f*_t_ [MPa]
Timber	GL24h	11,100 ± 1400 (*n* = 8)	35.0 ± 5.2	4.86 ± 0.7	443 ± 23	11.5 ± 0.7	-
	LVL48p	12,600 ± 260 (*n* = 5)	51.7 ± 1.3	5.99 ± 0.2	493 ± 5.3	9.6 ± 0.2	-
	LVL80p	16,800 *	81.7 *	8.72 *	841 ± 17	7.2 ± 0.6	-
Adhesive	EP1	6300	-	-	-		24.6 ± 6.8
	EP2	5400 ± 110	-	-	-	-	45.0 ± 1.4
	PUR	5000 ± 490	-	-	-	-	45.5 ± 0.7

* Determined from 5%-quantiles with COV = 5% and *k*_s_(*n*) = 1.645.

**Table 2:** Overview of test campaign with series designation and sample size.

	Material	Surface	EP1	EP2	PUR
Geometry 1 (vertical steel plate)	GL24h + S355	galvanized	V-RS-G-EP1 *n* = 2	V-RS-G-EP2 2	V-RS-G-PUR 1
		blasted	-	V-RS-B-EP2 1	-
	LVL48p + S420	galvanized	V-HS-G-EP1 2	-	V-HS-G-PUR 1
		blasted	V-HS-B-EP1 2	-	-
Geometry 2 (horizontal steel plates)	GL24h + S355	galvanized	H-RS-G-EP1 2	H-RS-G-EP2 2	H-RS-G-PUR 1
		blasted	-	H-RS-B-EP2 1	-
	LVL80p + S420	galvanized	H-HS-G-EP1 2	-	-
		blasted	H-HS-B-EP1 2	-	-

**Table 3:** Second moment of inertia.

	Material	Referred Second Moment of Inertia [×10^6^ mm^4^]		$\mathrm{Bending}\;\mathrm{Stiffness}\;\bar{EI}$ [×10^10^ Nmm²]
		*I_y,ref,T_*	*I_y,ref,S_*	
Geometry 1 (vertical steel plate)	GL24h + S355	62.9	3.31	69.5
	LVL48p + S420	58.8	3.53	74.1
Geometry 2 (horizontal steel plates)	GL24h + S355	107	5.64	118
	LVL80p + S420	79.6	6.38	134

**Table 4:** First moment of inertia [×10^4^ mm³].

	Material	Referred First Moment of Inertia [×10^4^ mm^3^]		
		*S_y_*_,_ *_ref_*_,__1,*T*_	*S_y_*_,_ *_ref_*_,__2,*T*_	*S_y_*_,_ *_ref_*_,__3,*T*_
Geometry 1 (vertical steel plate)	GL24h + S355	69.2	12.3	-
	LVL48p + S420	64.2	12.3	-
Geometry 2 (horizontal steel plates)	GL24h + S355	99.4	87.8	14
	LVL80p + S420	74.1	62.6	14

## Data Availability

Data is contained within the article.
